# Absence of clinical relationship between oxidized low density lipoproteins and diabetic peripheral neuropathy: a case control study

**DOI:** 10.1186/1476-511X-13-32

**Published:** 2014-02-12

**Authors:** Alma Rosales-Hernandez, Audrey Cheung, Peter Podgorny, Cynthia Chan, Cory Toth

**Affiliations:** 1 Department of Clinical Neurosciences, The Hotchkiss Brain Institute, and the University of Calgary, Calgary, AB, Canada; 2 Department of Clinical Neurosciences, HMRB 155, Foothills Hospital, University of Calgary, Hotchkiss Brain Institute, 3330 Hospital Dr. NW, T2N 4 N1 Calgary, AB, CANADA

**Keywords:** Type 2 diabetes mellitus, Cholesterol, Oxidized low density lipoprotein, Diabetic neuropathy, Diabetic neuropathic pain

## Abstract

**Background:**

The pathophysiology of diabetic peripheral neuropathy (DPN) is complex and uncertain. A potential comorbidity in diabetes mellitus (DM) that may contribute to greater severity of DPN is a lipid disorder, such as with elevated cholesterol, low density lipoproteins or triglycerides. Oxidized low density lipoprotein (oxLDL) is a form of cholesterol that exerts direct toxic effects and contributes to pathogenicity through ligating a receptor called lectin-like receptor (LOX-1).

**Methods:**

We examined plasma oxLDL levels in cohorts of patients with DPN with neuropathic pain (NeP), DPN patients without NeP, DM patients without DPN, patients with idiopathic peripheral neuropathy, and control subjects without DM or neuropathy. Our outcome measure was extent of oxLDL elevation, measured as fasting with Enzyme-Linked ImmunoSorbant Assay (ELISA) studies. Severity of diabetes was assessed using hemoglobin A1C measurements. Neuropathic severity was measured with the Utah Early Neuropathy Score (UENS). We hypothesized that DPN presence would be associated with oxLDL elevations.

**Results:**

A total of 115 subjects (47 with DPN and NeP, 23 with DPN without NeP, 12 with diabetes only, 13 with idiopathic peripheral neuropathy, and 20 control subjects without diabetes or neuropathy) were studied. Duration of diabetes and diabetic glycemic measures were similar between populations with DM. Severity of DPN was similar between cohorts with DPN and NeP and DPN without NeP. Plasma oxLDL levels were similar between all cohorts, without any elevation in the presence of DM noted in any cohort with DM.

**Conclusions:**

oxLDL levels are not different in patients with DPN, and their lack of greater presence suggests that any pathogenic role in human DPN is likely limited.

## Introduction

Diabetic polyneuropathy (DPN) is a common and disabling complication of human diabetes mellitus (DM) seen in up to 50% of patients, leading to sensory, motor and/or autonomic dysfunction [1]. There are several potential mechanisms postulated to contribute to development of DPN, including: 1) excessive sorbitol-aldose reductase pathway flux; 2) protein kinase C (PKC) isoform(s) overactivity; 3) increased oxidative and nitrative stress; 4) microangiopathy; 5) accumulation of advanced glycation end products (AGEs) and interaction with their receptor (RAGE); and 6) failure of neurotrophic support. However, studies to date have failed to show therapeutic benefit when many of these pathways have been targeted clinically.

A common association with DM is the presence of a lipid disorder, or hyperlipidemia. This may include hypertriglyceridemia, elevated cholesterol, or may occur as part of a metabolic syndrome. Although this is clearly a risk factor for cerebrovascular and cardiovascular disease, hyperlipidemia may also influence the peripheral nervous system, possibly correlating with progression of DPN [2] or human immunodeficiency virus-related peripheral neuropathy [3]. Complicating matters further is the possibility that statin medications used for treatment of hyperlipidemia may also contribute to peripheral neuropathy [4-6]. However, it remains unclear if either hyperlipidemia or its treatment has a true relationship with initiation or progression of peripheral neuropathic conditions.

An important form of cholesterol is called oxidized low density lipoprotein (oxLDL), which is known to have a critical function in the development of atherosclerosis. Along with its receptor, the lectin-like oxLDL receptor (LOX)-1 [7,8], both oxLDL and LOX-1 contribute to intracellular oxidative stress and inflammation injury within vascular tissues as well as in the immune system [9,10]. This may exist in an additive state with DM, as both oxLDLs and hyperglycemia increase LOX-1 expression [11,12]. In murine models, high fat ingestion leads to significantly increased plasma oxLDL levels in combination with morphological and functional evidence of peripheral neuropathy before hyperglycemia develops [13]. Also, oxLDLs contribute to oxidative stress and injury in dorsal root ganglia sensory neurons *in vitro* via a LOX-1 mechanism related to activation of nicotinamide adenine dinucleotide phosphate oxidase [13]. These data not only suggest that oxLDL and LOX-1 may contribute to DPN, but that they may also lead to development of peripheral neuropathy even in the absence of DM. This preclinical finding has not yet been confirmed in human studies.

We hypothesized that patients with DM and DPN would have higher levels of plasma oxLDL than patients with DM without DPN and as compared to control subjects. Furthermore, we postulated that patients with DPN subjected to neuropathic pain (NeP) would have greater oxLDL levels than those without NeP, based upon preclinical studies suggesting that curcumin, a molecule whose activity includes LOX-1 antagonism [14-16], relieves neuropathic pain including that associated with DPN [17-19]. As such, we performed a multi-cohort study to measure plasma oxLDL levels in adult patients with type 2 DM and control subjects for oxLDL levels to determine association with presence and severity of DPN. In addition, we recruited subjects with idiopathic peripheral neuropathy for comparison to the subjects with DPN. Should oxLDL be determined to be a risk factor for presence and greater severity of DPN, potential therapeutic pathways could be examined in the future.

## Results

### Patient and control subjects

A total of 128 potential subjects were screened for study involvement. Of these, 13 were excluded due to inability to perform phlebotomy (3 subjects), unclear presence of peripheral neuropathy (5 subjects), presence of possible peripheral neuropathy in control subjects (2 subjects), uncertain presence of NeP (1 subject with DPN), and inability to perform arranged blood work testing (2 subjects). Thus, a total of 115 subjects participated, including 20 healthy control subjects (16 women), 13 subjects with idiopathic peripheral neuropathy (7 women), 12 subjects with non-complicated DM (DM only) (6 women), 23 sub diagnosed with DM with DPN without pain (DPN-NoP) (7 women), and 47 subjects diagnosed with DM with DPN with persistent pain (DPN-P) (17 women) (Table 1). Of the assessed subjects with DPN, no other causation for peripheral neuropathy was present. Subjects with idiopathic peripheral neuropathy had no identifiable cause after detailed testing.

**Table 1:** Subject characteristics

	**Control subjects (n = 20)**	**Idiopathic neuropathy subjects (n = 13)**	**Diabetes mellitus subjects (n = 82)**		
			**Diabetes mellitus only (DM Only) (n = 12)**	**Diabetic peripheral neuropathy without neuropathic pain (DPN-NoP) (n = 23)**	**Diabetic peripheral neuropathy with neuropathic pain (DPN-P) (n = 47)**
Age	58.5 ± 12.7 years	60.7 ± 15.2 years	53.6 ± 14.4 years	61.1 ± 10.0 years	59.8 ± 10.3 years
Gender (males)	4/20 (20%)	6/13 (46%)	6/12 (50%)	16/23 (69%)	30/47 (64%)
Duration of Diabetes Mellitus			7.2 ± 4.6 years	8.6 ± 3.6 years	9.1 ± 3.3 years
Duration of Neuropathic Symptoms				6.8 ± 4.2 years	7.1 ± 4.7 years
Concomitant Diabetic Complications Present (other than neuropathy) Concomitant Vascular Risk Factors:			1/12 (8%)	6/23 (26%)	10/47 (21%)
Hypertension	3/20 (15%)	3/13 (23%)	8/12 (67%)	17/23 (74%)	21/47 (45%)
Hyperlipidemia	4/20 (20%)	2/13 (15%)	8/12 (67%)	15/23 (65%)	26/47 (55%)
Family History of Peripheral Neuropathy	0/20 (0%)	0/13 (0%)	0/12 (0%)	0/23 (0%)	0/47 (0%)
Concomitant Statin Use	4/20 (20%)	2/13 (15%)	7/12 (58%)*	14/23 (61%)*	23/47 (49%)*
Hemoglobin A1C (%) (4.3-6.1%)	5.2 ± 0.5	5.3 ± 0.8	7.4 ± 0.9*	7.4 ± 1.3*	7.8 ± 1.8*
Total Cholesterol (4.2-5.2 mmol/L)	4.5 ± 0.7	4.8 ± 0.8	3.8 ± 0.7	4.1 ± 1.0	4.2 ± 1.1
Triglycerides (0.6-2.3 mmol/L)	1.2 ± 0.4	1.4 ± 1.4	1.3 ± 0.7	1.6 ± 0.8	2.0 ± 1.1*
High Density Lipoprotein (>0.9 mmol/L)	1.5 ± 0.5	1.5 ± 0.5	1.3 ± 0.4	1.2 ± 0.3	1.3 ± 0.7
Low Density Lipoprotein (2.2-3.4 mmol/L)	2.9 ± 0.7	3.1 ± 1.1	2.2 ± 0.7	2.3 ± 0.9	2.3 ± 1.4
Toronto Clinical Neuropathy Score	0.5 ± 1.1	8.3 ± 3.6	0.6 ± 1.1	10.8 ± 4.0	14.5 ± 5.3^ψ^
Utah Early Neuropathy Score	0.6 ± 1.9	7.9 ± 3.9	0.5 ± 1.0	10.4 ± 4.7	14.3 ± 7.8^ψ^

Values shown are means ± standard deviations. *indicates p < 0.05 (one way ANOVA testing) for comparison of cohorts as compared to the control cohort. ^ψ^indicates p < 0.01 between cohorts DPN-NoP and DPN-P (one way ANOVA testing).

There was a significant difference in gender representation between control subjects and DM subjects (χ^2^ = 12.24, p < 0.001), but other comparisons had similar gender representations for analysis (χ^2^ = 0.22-1.09, p = 0.30-63). There were no significant differences in age representation between groups for comparison (ANOVA, p = 0.45). For DM subgroups, there were no significant differences in durations of DM (ANOVA, F = 0.45-0.87, p = 0.35-57) or with the presence of other diabetic complications (χ^2^ = 0.12-0.76, p = 0.44-0.72). There were no differences in statin use between DPN subjects and DM only subjects (χ^2^ = 0.12, p = 0.72), or for DPN-P subjects as compared to DPN-NoP subjects (χ^2^ = 0.88, p = 0.35). However, statin use was much more prevalent in DPN subjects as compared to idiopathic neuropathy subjects (χ^2^ = 6.18, p = 0.01), and in DM subjects versus control subjects (χ^2^ = 7.31, p = 0.01).

### Primary outcome measure–oxidized low density lipoprotein levels

**Figure 1:**
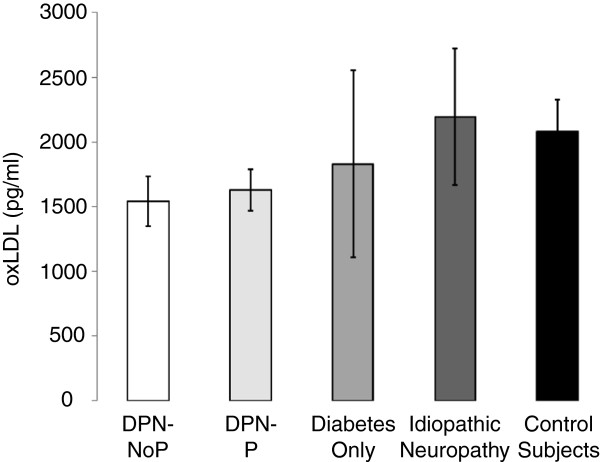
**Mean plasma oxLDL levels are demonstrated for each group.** There were no significant differences detected between any groups for any of the relevant comparisons. Error bars denote standard error of the mean (SEM).

No significant difference in oxLDL levels could be detected between all subjects with DPN versus subjects with DM only (ANOVA, 1633 ± 121 pg/ml vs. 1829 ± 721 pg/ml, F = 0.22, p = 0.64) (Figure 1). DPN-P subjects had similar levels of plasma oxLDL as DPN-NoP subjects (ANOVA, 1703 ± 160 pg/ml vs. 1541 ± 195 pg/ml, F = 0.45, p = 0.51). With comparison of all subjects with DPN and subjects with idiopathic peripheral neuropathy, levels of oxLDL were again similar between groups (ANOVA, 1633 ± 121 pg/ml vs. 2149 ± 525 pg/ml, F = 2.73, p = 0.11). In fact, those subjects with idiopathic peripheral neuropathy had a non-significant higher level of oxLDL despite the absence of DM. Finally, there was no significant difference in oxLDL levels between all subjects with DM as compared with control subjects (ANOVA, 1675 ± 170 pg/ml vs. 1913 ± 232 pg/ml, F = 0.64, p = 0.42).

### Secondary outcome measures

For DM subgroups, there were no significant differences in HbA1C levels between all DPN and DM only groups, as well as for DPN-NoP and DPN-P comparisons (ANOVA, F = 0.75-0.96, p = 0.33-68). HbA1C levels were significantly different between the entire DM group and control subjects (ANOVA, F = 38.8, p < 0.0001), as well as for DPN subjects when compared to idiopathic peripheral neuropathy subgroup (ANOVA, F = 21.9, p < 0.0001).

For all relevant comparisons, there were no statistically significant differences in total cholesterol (multiple ANOVAs, F = 0.04-3.74, p = 0.06-0.85) or HDL (multiple ANOVAs, F = 0.01-2.01, p = 0.16-0.91) between subgroups. Triglycerides were higher in all subjects with DM as compared to control subjects (ANOVA, F = 4.9, p < 0.05), but other relevant comparisons showed similar levels of triglycerides between groups (multiple ANOVAs, F = 1.49-3.38, p = 0.06-0.23). Also, and interestingly, low density lipoprotein (LDL) levels were lower in DM subjects as compared to control subjects (ANOVA, F = 4.4, p = 0.04) and were also lower in all DPN subjects as compared to idiopathic neuropathy subjects (ANOVA, F = 4.2, p = 0.04); there were no differences in LDL levels between DPN subjects as compared to DM only subjects (ANOVA, F = 0.1, p = 0.74) or for DPN-NoP subjects as compared to DPN-P subjects (ANOVA, F = 0.0, p = 0.98).

### Associations between oxLDL levels and other variables of interest

**Figure 2:**
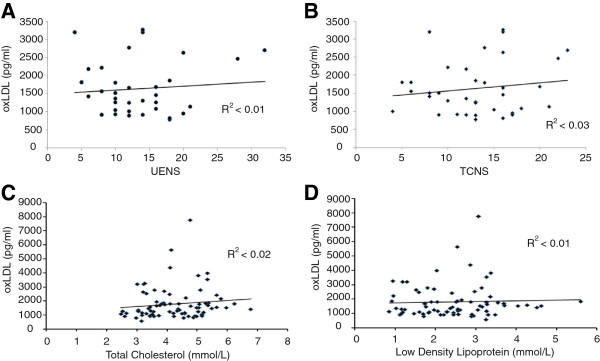
**Linear relationships were sought between oxLDL and clinical scores of peripheral neuropathy as well as for laboratory measurements for lipids.** For all subjects with DPN, there was no significant relationship between plasma oxLDL levels and the UENS **(A)** or TCSS **(B)**. For all subjects with DM, there was no significant association between plasma oxLDL levels and total cholesterol **(C)** or low density lipoprotein levels **(D)**. Linear regression was performed, with R^2^ values presented for each association sought.

Potential relationships were sought for oxLDL levels (independent variable) and TCSS, UENS, total cholesterol, and low density lipoprotein levels (dependent variables) (Figure 2). For all subjects with DPN, there was no relationship between oxLDL levels and either of TCSS (R^2^ < 0.03, p = NS) or UENS (R^2^ < 0.01, p = NS). For all DM subjects, there was no relationship between oxLDL with either total cholesterol (R^2^ < 0.02, p = NS) or low density lipoprotein (R^2^ < 0.002, p = NS). Finally, two post-hoc tests were performed. First, to examine the impact of statin medication use, the oxLDL levels of DM subjects taking statin medications were compared to those of DM subjects not taking statin medications; there was no significant difference between these subgroups (ANOVA, F = 1.20, p = 0.28). Second, linear regression was performed to compare total cholesterol and low density lipoprotein levels–there was a significant relationship for measures obtained in all subjects (R^2^ = 0.37, F = 42.7, p < 0.01).

### Association with clinical severity of neuropathy and diabetic complications

With clinical severity of peripheral neuropathy as the dependent variable, there was no relationship between UENS severity and oxLDL levels (R^2^ < 0.01, p = NS), HbA1C (R^2^ = 0.05, p = 0.20), total cholesterol (R^2^ = 0.01, p = NS), triglyceride levels (R^2^ < 0.01, p = NS), and LDL levels (R^2^ = 0.02, p = NS). Likewise, there were no significant relationships between TCSS severity and oxLDL levels (R^2^ < 0.01, p = NS), HbA1C (R^2^ = 0.03, p = NS), total cholesterol (R^2^ = 0.03, p = NS), triglycerides (R^2^ < 0.01, p = NS), and LDL levels (R^2^ = 0.03, p = NS). Finally, there was no association between the number of recorded diabetic complications and oxLDL levels (R^2^ = 0.01, p = NS).

## Conclusions

We sought to determine if oxLDL levels were heightened in patients with DM-mediated DPN and related to the presence of neuropathic pain in DPN-P patients. Our results indicate that we were unable to determine any relationship between plasma oxLDL levels and the presence of DPN or the severity of DPN. There was also no significant increase in plasma oxLDL levels in the presence of DM as compared to a non-diabetic control state. Furthermore, there was no detected worsening in clinical severity of neuropathy with higher levels of oxLDL, HbA1C, total cholesterol, triglycerides, or LDL levels. Recently reported studies have also demonstrated a lack of elevated oxLDL levels in patients with DPN or DM without DPN when compared to control subjects [20]. Although prior studies indicate a positive relationship between any diabetic microvascular complication and abnormalities in LDL, triglycerides, and non-HDL cholesterol [21] with similar overall cholesterol results, it remains unclear regarding any association between lipid status and presence of peripheral neuropathy. Thus, although preclinical studies have identified potential mechanisms by which oxLDL may contribute to neurodegeneration, particularly in the presence of hyperglycemia related to DM, our clinical study did not identify any particular relationships.

The present findings should not be seen to discourage against further investigations of other methods by which hyperlipidemia could contribute to neurodegeneration in DM patient populations. There is clinical evidence that triglyceridemia contributes to progression of already existing DPN [2], while each of total cholesterol, low-density lipoprotein and triglyceride levels have been positively associated with increasing cumulative incidences [22] or greater severity [23] of diabetic peripheral neuropathy. Furthermore, a low high density lipoprotein level may also relate to presence of DPN [24]. The mechanisms by which these forms of cholesterol may impact upon peripheral nerve function and structure are unclear. It is also difficult to simulate these human relationships in animal models; for example, mice have quite low levels of low density lipoproteins, while most cholesterol is transported by high density lipoproteins [25], distinct from the human situation. Despite this issue, wildtype mice fed high fat diets develop elevated plasma oxLDL levels [13]. Peripheral nerve studies in transgenic mice featuring highly increased plasma cholesterol are untenable due to excessive short term mortality [25]. In wildtype mice, a high fat diet plays an important role in the severity of experimental DM [26,27], and it is even possible that a high fat diet may lead to peripheral neuropathy in the absence of DM [28]. Thus, there is sufficient preclinical and clinical data to suggest that an association exists between lipid disorders and peripheral neuropathy, but the potential mechanism has remained elusive.

Further complicating our ability to understand relationships between lipid disorders and peripheral neuropathy is the controversial relationship of frequently prescribed statin medications used for lipid disorder management. Statins lower cholesterol through inhibition of 3-hydroxy-3-methylglutaryl coenzyme A (HMG-CoA) reductase. Statin use is extremely common [29,30], particularly in diabetic subjects; in our study, over 50% of DM subjects were taking statins. There are a number of case series and case-control studies [6,31-33] along with a recent cross-sectional study [30] suggesting a relationship between statin use and peripheral neuropathy presence. However, there are also some contrasting reports, suggesting that statins may have a beneficial effect upon peripheral neuropathy in humans (limited to hypotheses) [34,35] and rodents [36-38]. Nevertheless, given the absence of a plausible mechanism by which statins could induce peripheral neuropathy, the effect of statins upon peripheral nerves remains unclear. In our study, the use of statins was significantly more common in the diabetic cohorts, which likely contributed to relatively depressed LDL levels relative to Control Subjects and Idiopathic Neuropathy Subjects. This is a confounder that certainly impacts upon our results as statins certainly will decrease oxLDL levels [39,40] in our diabetic cohorts. This obstacle is difficult to overcome in clinical studies, as the great majority of diabetic patients now receive statin therapies. The washout of statin medications would likely be required in future studies of experienced DM patients to determine natural oxLDL levels; alternatively, investigation of newly diagnosed DM patients prior to interventions may assist in future determinations.

We chose to examine oxLDL due to its potential for neurotoxicity. Known to have a critical function in the development of atherosclerosis [7,41,42] along with its receptor, the lectin-like oxLDL receptor (LOX)-1 [8], its impact upon non-neural tissues has great substantiation. The receptor LOX-1 has non-constitutive but dynamically inducible expression [43] driven by both oxLDLs and hyperglycemia [11,44]. Along with oxLDL, LOX-1 contributes to intracellular oxidative stress and inflammation injury [9,10]. In vitro, oxLDLs contribute to oxidative stress and injury in DRG neurons via a LOX-1-mediated mechanism related to activation of NAD(P)H oxidase [13]. These data not only suggest that oxLDL/LOX-1 may contribute to DPN, but that they may also lead to development of peripheral neuropathy even in the absence of DM. Dietary alterations may also play roles: high-fat feeding in murine models significantly increases plasma oxLDL as well as contributing to morphological and functional evidence of peripheral neuropathy prior to development of hyperglycemia [13]. Therefore, although our study failed to identify higher levels of oxLDL in human subjects with DPN (or even only with DM), there is evidence to suggest that elevated oxLDL levels can be neurotoxic.

Our findings are presented with limitations to be considered. Although we used a standard ELISA method to measure oxLDL levels, there are different methods that may be favored in some circumstancses [45]. Also, other methods of analyses including in vivo measurement [46] and ex vivo oxidation assays [47] exist. In some cases, serum oxLDL levels [48,49] are measured instead of plasma oxLDL levels [42,50]–it remains unclear which technique may be most accurate. Studied subjects were volunteers and were not randomly selected from a population with type 2 DM with or without DPN and with or without accompanying NeP. Our sample size was not based upon a pre-determined power analysis. Patients with type 1 DM were excluded due to potential differences in the pathophysiology of neurodegeneration and to maintain a more homogeneic patient population. As stated, gender matching between groups was not complete as female subjects were overrepresented in the control group. Also, statin use was much more common in DM patients, although its use was not associated with significant changes in measured oxLDL levels. The use of pharmacotherapies for management of DM and chronic pain related to DPN may have impacted upon oxLDL measurements in uncertain ways. Although total cholesterol and low density lipoprotein levels were positively associated, there was no association found between lipid measurements and plasma oxLDL levels. Clinical severity of neuropathy failed to correlate with types of lipids measured or with HbA1C levels. Although higher HbA1C levels are associated with risk of subclinical neuropathy [51] and electrophysiological parameters [52], HbA1C is not necessarily an independent predictor of diabetic neuropathy although it is clearly associated with other microvascular complications [53,54]. However, in prior studies examining DPN populations, the clinical severity of DPN based upon the UENS was positively associated with age, duration of diabetes, height, weight and HbA1C [55]. Our study population was small, but the lipid status and HbA1C values are similar to similar populations of DPN patients studied [55]. We did not perform measurement of other markers for oxidative stress such as glutathione, malondialdehyde, or homocysteine, which may be elevated in DPN patients [56] and could have provided comparison to measured oxLDL and HbA1C levels. Finally, the individual group sizes were small after categorization, and we cannot rule out the possibility of type II errors contributing to the lack of statistical associations determined–future larger studies may be more forthright. However, there was a distinct lack of relationship between oxLDL levels and DPN manifestations found amongst studies performed to date.

Our results demonstrated no visible associations between oxLDL levels in DM subjects and the presence or severity of DPN. The results of our study do not exclude the possibility of elevated levels of oxLDL at the level of the peripheral nerve itself contributing to DPN [13,57]; we did also not study the role of the LOX-1 receptor in our population. As a result, further more invasive studies may determine other potential findings related to oxLDL. Future studies may examine other aspects of lipid disorders for relationship with complications of DM, including DPN. Although our hypothesis of plasma oxLDL mediating greater presence and severity of DPN in subjects with DM was not verified, it is possible that our results may be confounded by some of the factors discussed above. Factors other than plasma oxLDL levels should be sought to determine causative or contributory factors in the development of DPN.

## Methods

### Participant recruitment

Ethical approval for this study was received from the University of Calgary Centre for Advancement of Health. Recruitment of subjects occurred from March 2010 until January 2013 using poster recruitment within the Neuromuscular, Neuropathic Pain and Endocrinology Clinics at the University of Calgary. All subjects provided informed written consent prior to their involvement.

Subjects with a diagnosis of pre-existing type 2 DM were included following confirmation of diagnosis based on Canadian Diabetes Association guidelines [58]. Briefly, this required verification of two prior separate positive laboratory results from any of the following: fasting glucose results of ≥ 7.1 mmol/L (126 mg/dL) or two oral glucose tolerance tests leading to a 2 hour serum glucose of ≥ 11.1 mmol/L (200 mg/dL), or a random glucose of ≥ 11.1 mmol/L (200 mg/dL) at any time. The age of diagnosis of DM and the duration of symptoms of DPN (if present) were recorded. During patient history, subjects found to have DPN were also assessed for the presence of other systemic illnesses, prior or current alcohol dependence (based upon Diagnostic and Statistical Manual of Mental Disorders (DSM)-IV criteria), toxin and medication exposures, and family history of neuropathy was documented to assess and exclude other potential causes of peripheral neuropathy. The intake of anti-DM pharmacotherapies, statins used for management of hyperlipidemia, and other medications (including those used for chronic pain management) were recorded. The presence of diabetic complications other than that of DPN and other vascular risk factors were also noted.

Subjects with a diagnosis of idiopathic peripheral neuropathy were included based upon negative laboratory testing for other potential causes of peripheral neuropathy, including but not limited to laboratory testing as previously described [59]. Control subjects were age-matched and culled from non-diabetic friends and relatives of patient subjects, as well as from staff working at each of the clinics participating in this study.

### Phlebotomy and oxidized low density lipoprotein testing

All subjects, fasting for a minimum of 12 hours, underwent phlebotomy. This whole blood sample, stored in a plasma tube with lithium heparin and gel (plastic tube, light green top, Vacutainer, Becton Dickenson), was inverted gently over the following 30 minutes. Then, the whole blood sample was placed on ice for another 30 minutes until it was submitted to refrigerated centrifugation within a 1 hour time period after phlebotomy, performed at 1500 g for 10 minutes. The supernatant was collected as plasma–this liquid component was transferred to a 1.5 ml Eppendorf tube while at 4°C. Following this, plasma was stored at −80°C for 1-3 months until further testing was performed. Plasma oxLDL was measured by Enzyme-Linked ImmunoSorbant Assay (ELISA) (Uscn Life Science Inc., Wuhan, China) following their provided specifications.

Plasma samples were diluted 1000X using phosphate buffered saline and standards were prepared according to manufacturer’s instructions. 100 μL of standard and diluted samples were added to wells pre-coated with a monoclonal antibody specific for oxLDL, with incubation occurring for 2 hours at 37°C. Following this, a biotin-conjugated polyclonal antibody specific for the oxLDL antibody was added. After successive washings, an avidin conjugated to horseradish peroxidase was added to each well and incubation followed again. Then, a 3,3′,5,5′-Tetramethylbenzidine substrate solution was added, providing a colorimetric change after interaction with oxLDL. The enzymatic substrate reaction was terminated with the addition of sulphuric acid. Colorimetric change was measured immediately using a spectrophotometer with detection at 450 nm. The concentration of human OxLDL in plasma was determined by comparing the optical density of the samples to the standard curve obtained by similar measurement of the standards.

Within one month of assessment, additional fasting blood was evaluated for HbA1C, total cholesterol, triglycerides, high density lipoprotein (HDL), and low density lipoprotein (LDL) (Calgary Laboratory Services, Calgary, AB, Canada) for all subjects.

Patients were excluded if potential causes for peripheral neuropathy other than DM were identified, if they had impaired glucose tolerance only, or if they had a juvenile onset of DM or frank requirement for insulin at diagnosis (i.e. possible type 1 DM). Lastly, subjects were excluded if they refused concurrent laboratory testing.

### Subject assessment and allocation

Each subject underwent a neurological examination that permitted scoring for presence and severity of peripheral neuropathy using the Toronto Clinical Scoring System (TCSS) [60] and the Utah Early Neuropathy Scale (UENS) [61]. This was performed by an unblinded Neurologist subspecializing in Neuromuscular Diseases. After clinical scales were completed, subjects were categorized to have either no peripheral neuropathy (control subject or DM only subject, both TCSS ≤ 3 and UENS ≤ 3) or to have peripheral neuropathy (Idiopathic Neuropathy Subject or DPN subject both TCSS > 5 UENS > 6). Those subjects with a TCSS score of 4-5 and/or a UENS score of 4-6 were excluded due to uncertainty regarding the presence or absence of peripheral neuropathy. As well, control subjects were excluded from participation if TCSS > 4 and/or UENS > 3. We further categorized subjects with DPN as having NeP using the question “Do you have pain on a daily or near daily basis?”. If subjects answered “yes”, then they were asked to complete the Douleur Neuropathique 4 questionnaire (DN4Q), which categorizes pain as neuropathic or non-neuropathic in nature with good sensitivity (82.9%) and specificity (89.9%) [62]. Subjects scoring ≥4 on the DN4Q were categorized to have DPN with NeP (DPN-P), while subjects denying any pain or discomfort (answering “no”) were categorized as DPN without NeP (DPN-NoP). If subjects admitted to having pain, but had a score of <4 on the DN4Q, they were excluded from further participation due to uncertainty regarding the nature of experienced pain. In order for subjects to be categorized as DPN-P, their pain must be chronic, having persisted for ≥3 months with pain severity estimated to be ≥40 mm on the 100 mm visual analog scale (VAS) of the Short-Form-McGill Pain Questionnaire; otherwise, these subjects were excluded due to uncertainty of classification as DPN-P or DPN-NoP.

### Statistical analysis

Group equivalence for participant age, duration of type 2 DM, duration of DM and DPN symptoms, statin use, presence of other diabetic complications, and hemoglobin A1C were compared by one-way ANOVA testing; gender was compared by chi-square testing using Fisher’s exact test to calculate two tailed p values. Chi-square testing was also used to compare the presence of other diabetic complications. The primary outcome measure was a mean difference in plasma oxLDL levels. To test for statistical differences in plasma oxLDL levels between groups, we performed individual ANOVA testing between the following cohorts of interest: 1) subjects with DPN (DPN-P and DPN-NoP subjects) compared with DM only subjects; 2) subjects with DPN-P compared with DPN-NoP subjects; 3) subjects with DPN (DPN-P and DPN-NoP subjects) compared with idiopathic peripheral neuropathy subjects; and 4) all subjects with DM (DM only, DPN-P, and DPN-NoP subjects) as compared with control subjects. We then assessed for statistical differences in secondary outcome measures including HbA1C, total cholesterol, triglyceride, high density lipoprotein, and low density lipoprotein levels between the aforementioned groups. We also performed linear regression analyses to determine relationships between oxLDL levels (independent variables) and TCSS, UENS, total cholesterol, and low density lipoprotein levels using pair-wise Bonferroni corrections for multiple comparisons. A post-hoc analysis was performed with linear regression analysis of the number of diabetic complications present (each of neuropathy, retinopathy, nephropathy, cardiovascular disease, cerebrovascular disease) as the independent variable and oxLDL levels as the dependent variable. Finally, in DPN subjects, post-hoc linear regression analyses were performed using severity of DPN (TCSS and UENS) as a dependent variable and independent variables of oxLDL levels, Hemoglobin A1C, total cholesterol, triglycerides, and LDL levels. Values were expressed as mean ± standard deviation or mean ± standard error as specified.

## Competing interests

The authors declare that they have no competing interests.

## Authors’ contributions

AR-H performed all ELISA testing. AC and PP contributed to data obtainment. CC assisted with organization of scheduling, data obtainment, and phlebotomy and preparation of human blood samples for testing. CT oversaw the study, approved and edited the protocol, assisted with analysis of data, performed data obtainment, performed history and examination for all subjects and composed the manuscript. All authors reviewed and contributed to the editing of the manuscript. All authors read and approved the final manuscript.

## Authors’ information

CT is a Neuromuscular Neurologist studying diabetic peripheral neuropathy.
